# Practical Remarks on the Diseases of the Skin, on the External Signs of Disorder, and on the Constitutional Peculiarities during Infancy and Childhood

**Published:** 1838-10

**Authors:** 


### Art. IX.

1. *Practical Remarks on the Diseases of the Skin, on the external Signs of Disorder, and on the Constitutional Peculiarities during Infancy and Childhood.* By Walter C. Dendy, Surgeon to the Royal Infirmary for Children, &c.—*London*, 1837. 8vo. pp. 153.
2. *A short Treatise on the external Characters, Nature, and Treatment of the different Forms of Porrigo, or Scalled Head, and Ringworm.* By Walter Dick, m.d.—*Glasgow*, 1838. 8vo. pp. 58.

Both these works profess to be rather practical than scientific; and, considering the great and often superfluous learning which we have to encounter in the volumes that come before us, we are not sorry to meet with books containing nothing which has not a direct relation to therapeutics; a branch of medicine which, strange to say, certain medical writers think beneath their notice. The public, however, take a more utilitarian view in this case; and we believe that few medical men have ever obtained a really beneficial reputation by writings which did not comprise valuable therapeutics, or discoveries from which improvement in the treatment of diseases could not be very directly derived.

I. Mr. Dendy has drawn from his opportunities as surgeon to the Royal Infirmary for Children valuable materials for various disquisitions on the diseases of infancy; a subject, of which we all feel the difficulty and importance. The first essay in the present volume, on the Constitution of Infants, contains, as might be supposed, much that is familiar to the experienced practitioner: it also comprises some remarks and suggestions, which, if not quite original, are at least less generally known, and are well worthy of being recorded. The following observations are of this kind:

"When the physiologist begins to explain the phenomena of organic life, he selects the animal in which the organization is the most simple; so the constitutions of young children are the most favorable for the study of disease, because they are usually marked by greater simplicity, unmodified by alarm regarding the result of their disease, or by sexual influence, or by mental emotion or disquietude, or by the physical changes resulting from the wear and tear of body. They are, above all, uninfluenced by the alterations of structure produced by repeated and varied disease; for acute attacks, if not checked very early, soon disorganize and change the normal condition of some important organs, forming thus what is termed 'a weak or delicate point;' becoming often, in after life, the focus or seat of disease." (p. 3.)

Notwithstanding this greater simplicity in the form of diseases in infants, compared with adults, our want of that portion of symptoms (and we all know how considerable a portion it is,) derived from the patient's description of his own feelings, renders them difficult of investigation. Mr. Dendy appears to us to estimate far too low the verbal information derived from patients, when he speaks of it as "expressions, which, from the imperfection of language, and the adoption of figurative terms, are not always so decisive and illustrative as we think." Admitting this, we still think that, by a little cross-examination, the physician need never have any difficulty in reducing the patient's language to its just value: for a vague term, such as "*bilious*," for instance, he may obtain a precise description of the patient's feelings, and be enabled to correct such errors as are conveyed by the word "weakness," when employed to represent obscure local pain, or to describe febrile oppression.

To obviate the difficulty arising from the want of articulate speech, the author endeavours to make us correct interpreters of the natural signs of disease—the instinctive language of complaint during pain, the expression of the features, the attitude and action of the limbs. The varieties of these natural signs are drawn concisely, but with graphic power; and we earnestly recommend a perusal of the sketch. The following extract is part of the description of the language of complaint during pain:

"*Screaming* is a violent effort, indicating vigour, and is usually heard in the early or acute period of disease. The face is flushed and the veins turgid, as the effort impedes the return of blood from the head. When it is protracted, becomes shrill and piercing, and the heat of skin is increased, inflammatory action has probably commenced.

"In inflammation of the gum during dentition, &c., the scream will be more or less protracted; but in inflammation of the chest and belly, in which are seated the organs of breathing, the effort of screaming, induced by continual suffering, so much increases pain, that the child controls for some moments its expression; the screaming is by fits and starts. Local symptoms will direct us to the seat of pain. Intolerance of light and tossing of the head, to the brain; quick breathing, or panting, or cough, to the lungs; palpitation, to the heart; constipation or diarrhœa, to the bowels; nausea, to the stomach; and crowing, to the larynx." (p. 15.)

The section on Diseases of the Skin is by much the longest and most important of the work. The author, in a very sensible introduction to his classification of these diseases, illustrates from them the remark of Sydenham that "a disease is no more than a vigorous effort of nature to throw off morbific matter, and thus recover the patient." He disclaims, indeed, the implicit adoption of a humoral pathology; but is of opinion that cutaneous affections are the outlets and safety-valves of serious internal disorders, and that many resist our means of cure from an endeavour of nature to perpetuate relief to the inward disease causing the eruption. On the other hand, he states the fact, that the cure of these affections, when of long standing, often proves detrimental to the constitution. These opinions are confirmed by striking examples from other works, and supplied by the author's own observation. He admits, however, that the establishment of disease of the skin often reacts perniciously on the system, and that its extension to important parts may prove destructive.

Impressed with the truth of the connexion between certain internal diseased conditions and the outward and visible sign existing on the skin, the author has essayed to form what he terms a practical system of classification, founded on the causes of these diseases. He adopts the descriptions of Willan and Bateman; but employing (to use his own expression,) their delineations as an alphabet, and the classification according to causes as a language, he conceives he has thrown into a concise form all the information that science at present offers regarding cutaneous pathology.

The following is the arrangement according to causes:—The first division comprises diseases symptomatic chiefly of disorder of the alimentary canal, marked by increased cutaneous action, often by subacute or chronic inflammation. The diseases comprehended in this division are subdivided into those occurring during dentition or suckling,—viz. strophulus, lichen, prurigo, crusta lactea, impetigo; and those dependent chiefly on gastro-enteric irritation, which are roseola, erythema, eczema, urticaria, erysipelas, phlegmon, herpes, lepra, psoriasis, crinones, verruca follicularis, acne, sycosis, and porrigo. In the second division are placed diseases indicative of debility, marked by languid cutaneous action, often the sequelæ of acute disorder. These, again, are subdivided into affections depending on original debility of the system,—viz. pityriasis, icthyosis, elephantiasis, alopecia, shrivelled skin, ephidrosis; and those arising from derangement of the chylopoïetic function, which we learn are aphthæ, miliaria, ecthyma, rupia, pemphigus, anthracion, anthrax, purpura, nomé, struma, onychia maligna, cloasma. Of diseases consequent on specific infection we have likewise two subdivisions: the first the febrile, comprising rubeola, scarlatina, varicella, variola; the second the non-febrile, comprehending vaccinia, scabies, and syphilides. The fourth division contains diseases consequent on external and common irritation; which are, encausis, vesication from external irritants, pernio, paronychia, pterygion, verruca, clavus, intertrigo, rhagades, condyloma. The fifth division, or Maculæ, comprises nævus vascularis, lentigo, and tinge of argenti nitras; by which is meant the cerulean hue, deepening to purple or purplish black, produced by the *internal* use of this medicine.

It will be obvious that the propriety and value of this arrangement depend entirely on the correctness of the reference of the diseases comprised in the first two divisions to the causes assigned by Mr. Dendy. We are as much averse as possible to the practice, too common among certain writers, of emptying their case-books on the public: not only, however, would we have excused a few cases in the present instance, but we consider them absolutely necessary to justify the altered classification; for without them the profession, in adopting it, would be surrendering their conviction to Mr. Dendy's opinion instead of his arguments, which it would be quite unreasonable in him to expect. We think the juxtaposition of diseases in a nosological system, because of a resemblance in external character, without a correspondence in their essential nature, a matter of very trifling value: where the two conditions exist, as in the pyrexiæ of Cullen, they constitute a natural class. The latter condition, however, is infinitely the more important; and, had the author proved by the evidence of facts that his collocation of cutaneous diseases reposed on this basis, he would have received thanks for rescuing this branch of our art from the confusion and quackery in which, notwithstanding the labours of many eminent men, from Plenck to Rayer, it is still involved. We not only find a lack of credence on this point, but, as we read the book, positive objections to the classification occurred to us. Purpura, for instance, we find under the head of "diseases indicative of debility, marked by languid cutaneous action;" whilst the author, when mentioning the treatment of the disease, says, "where indications of congestion, of local pain, or *high action* especially, prevail, a small quantity of blood may be drawn in the recumbent position." Not only do we agree in this statement, but we may add, that we have as frequently found purpura associated with a sthenic as a debilitated state of the system, and have been compelled to resort not to small but copious bleedings, and every part of the antiphlogistic regimen, to subdue it.

Notwithstanding our distrust of Mr. Dendy's classification, we have been much gratified by the perusal of his remarks on many of the diseases. His practical good sense is throughout conspicuous, and nowhere more than in the following remark:

"The adoption of one principle in treatment will often relieve us from this dilemma. I mention it here generally, because, as a preliminary step, it is applicable to almost all cutaneous diseases, especially in childhood, whether common or specific, idiopathic or symptomatic: I allude to local depletion. By subduing common inflammation, which is concomitant with so many even contagious diseases, we produce almost a magical effect, by rendering a disease, before stubbornly resisting, directly yielding to local and even constitutional remedy." (p. 24,)

At the end of the work there is a formulary of local and constitutional remedies, which cannot fail to be valuable to the young practitioner, to whom we can conscientiously recommend this unpretending volume, as calculated to remove many obstacles in the difficult path of early practice.

II. Dr. Dick's work is of more limited range than Mr. Dendy's, but is written with the same laudable object, of disembarrassing the confused and illustrating the obscure in one of the most difficult departments of practical medicine. We know that professional reputations have sustained considerable injury in private families, and especially in schools, from erroneous or undecided opinions regarding the troublesome and loathsome disease which forms the subject of Dr. Dick's "Short Essay." We have seen no treatise upon it so well calculated to impart precise ideas to the young practitioner as this, and consequently to give decision and accuracy to any opinion he may express.

The author thinks, and very correctly, that the genus Porrigo should embrace only species displaying what may be termed the important characteristics of this troublesome family,—contagion, difficulty of cure, and alteration of the appearance of the hair. He, therefore, removes those comparatively slight diseases, Porrigo larvalis and P. favosa, from this genus, and places them with the Impetigines; and, consequently, the student of the disease has only to deal with P. lupinosa, P. furfurans, P. scutulata, and P. decalvans. As, moreover, he agrees with Mr. Plumbe in considering P. furfurans and P. scutulata one and the same disease, the number of species is thus still further reduced to three. This is the simplest classification of the disease that we have as yet seen; and, should general observation confirm the accuracy of Dr. Dick's opinion, he will deserve credit as a reformer in this department of dermatology.

A valuable portion of the treatise is the descriptive anatomy of the parts on which the author considers the *real* forms of porrigo to depend, —the hair and the sebaceous follicles of the scalp. This, however, we leave to be read in the work itself. To the former of these structures he refers ringworm, or P. scutulata and P. furfurans; to the latter, P. lupinosa. His reasons for referring the former disease to an affection of the pilous structure are thus stated:

"That ringworm consists primarily of subacute inflammation of a specific character, affecting the pilous cysts, and structure secreting the hair, we infer from the following circumstances:

"1st. The thin, bran-like incrustations so frequently seen surrounding the roots of the hairs, and consisting evidently of a morbid secretion from the pilous cysts.

"2d. The falling-off of the hairs from affected parts, and the towy, unhealthy appearance of those which remain.

"3d. In pulling out the hairs from affected parts, we have sometimes brought the bulbs along with them; and these never presented their usual or normal appearance.

"The disposition of morbid action to spread from the parts or tissues in which it originates to those adjacent, is well known; and in ringworm, as in other diseases, this extension of morbid action always occurs, to a greater or less extent. After the disease has continued for a considerable time, the whole thickness of the skin or affected parts is undoubtedly affected, or in a morbid state; but the arguments above adduced appear to me sufficient to authorize us to fix upon the pilous cysts and hair-bulbs as the parts primarily affected in ringworm.

"There seems to be something like a reciprocity of action, in health, between the vessels or organs secreting the cuticle and those secreting the hair; as we see the cuticle thin and delicate where the hairs are plentiful and strong, and *vice versâ*. I have been rather inclined to believe that the epidermic scurf of ringworm might partly result from an over-active state of the vessels secreting the cuticle at affected parts, in consequence of the check given by the disease to the secretion of healthy hair. In other and more extensive diseased states of the system, we see one organ performing, as it were vicariously, the action of another. But I must confess that I am not so much attached to the above notion as I was some time ago; and perhaps the phenomena of Porrigo decalvans are rather opposed to it." (p. 42.)

The author coincides in the opinion of Murray, and dissents from that of Duncan, Underwood, and Luxmore, in regarding the sebaceous follicles as the seat of the P. lupinosa. Baudelocque conceived that he had settled the question by adopting the opinion of Bichat, Meckel, and others, and denying the existence of sebaceous follicles in the scalp. Dr. Dick, however, cannot concur in this view, but describes the sebaceous follicles as really existing, placed at the exit of the hairs from the skin, and surrounding the summits of the pilous cysts. He derives a confirmation of his opinion regarding the seat of the disease from a preparation, which he thus describes:

"The preparation to which we above alluded, as confirmatory of the opinion of the disease being seated primarily in the sebaceous follicles, consists of a small portion of the scalp, inclosing an incipient lupine-like scab, taken from a subject who died of fever, and who had, for a considerable period before death, been affected with P. lupinosa. The scab is seen to be still covered by the cuticle: inferiorly, it appears to be inclosed in a membrane, and a hair enveloped in its double cyst, apparently in a healthy state, is seen passing through its centre. In short, the scab occupies exactly the situation of a sebaceous gland, and indeed appears to be merely a sebaceous gland distended by a concrete secretion. This view of the pathology of the disease appears to be confirmed by the fact that, when a lupine-like scab is removed, it is not succeeded by a similar one, but by one of an irregular form, without the peculiar cupped appearance. This arises, as we conceive, from the sebaceous follicle having been destroyed during the development of the primary scab." (p. 26.)

Dr. Dick's pathology of P. decalvans is not so distinct as his explanation of the symptoms of the other two species. He supposes that the principle which produces it may act upon the organs secreting the hair in such a manner as to produce a temporary atrophied or inactive state of them. But its true nature, he admits, is obscure; for it is often seen to continue a long time and afterwards disappear, either spontaneously or during the application of stimulant liniments, the hair sprouting up strong and healthy over the previously bald patches.

The author gives clear descriptions of the different forms of porrigo, but these we do not consider it necessary to copy, as they do not essentially differ from those found in Bateman and other elementary works. Regarding the causes of the disease, he thinks that it may arise spontaneously in the ill-fed and filthy; but contagion most commonly produces it. His own experience establishes the fact, that all the forms of real porrigo may arise from the same contagious principle; for when from one case of the disease other individuals of a family are infected, it not unfrequently happens that they are so in forms differing from the original one. The variety most frequently observed in this country, is the furfuraceous or true ringworm of the scalp; whilst in France, the P. lupinosa is the most common form.

The therapeutical suggestions of Dr. Dick are judicious. He is very anxious that this disease should be withdrawn from the domain of quackery, a feeling in which we concur with him; but till more decisive principles of treatment are laid down than are to be found in the pages of writers on this subject, those of Dr. Dick included, we fear that our practice must continue to be at least *empirical*. The vexed question of plucking out the hair he endeavours to settle in the negative, opposing to the authority of Plumbe and others the reasoning that the disease is not seated in the hairs themselves, but in the organs secreting them, and that no considerable benefit can accrue from their evulsion till the parts primarily affected are restored to a healthy state. The success of the depilatory ointments of the Frères Mahon he ascribes, not to their destroying the hair, but to their influence on the structure secreting it and on the cutaneous vessels. He recommends that loose hairs should be removed, that shaving the head should be practised where the condition of the scalp will allow it, and otherwise that the hairs should be cut short. The practice of Mr. Plumbe, which though severe we regard as founded on rational principles, smearing the affected parts with strong sulphuric acid, allowing it to remain for a minute or two, and then washing it off with tepid water, is approved of, and proposed to be extended from the form of the disease in which it was first adopted, ringworm, to porrigo lupinosa.

Dr. Dick extols poultices, and says that many cases have been entirely cured by them. This we can readily believe, and we are somewhat surprised that this result or other circumstances did not suggest to him the adoption of the practice so judiciously recommended by Mr. Dendy, that of subduing common inflammation. The quackery of which the author complains has consisted in treating this disease exclusively by irritants, the only question having been which of the tribe, tar, sal ammoniac, mercury, or sulphur, should predominate in the village nostrum. The name not the nature of the disease has been the object of the prescription. Only by more attention than has hitherto been paid to the local pathological condition, and to the constitutional state with which it is not unfrequently associated, a point which Dr. Dick too much neglects, can this disease be transferred from the domain of quackery to that of regular medicine. By leeches and poultices to the affected parts followed by some mild unctuous application, such as olive-oil or zinc ointment, whilst due attention is paid to the state of the digestive organs and the general system, more may sometimes be accomplished in a few days than by the entire class of irritants employed for months. There are, however, states of the disease for which stimulants are required; and the method of Mr. Plumbe we consider the most simple and efficacious representative of the class, and well suited to supersede the *farrago* by which medical men have been bewildered and patients tortured.

In conclusion we must express the opinion that Dr. Dick's "Short Essay" is the work of an industrious and enlightened physician, and a valuable contribution to this department of medicine.

---

